# Deep learning segmentation of organs‐at‐risk with integration into clinical workflow for pediatric brain radiotherapy

**DOI:** 10.1002/acm2.14310

**Published:** 2024-02-19

**Authors:** Lina Mekki, Sahaja Acharya, Matthew Ladra, Junghoon Lee

**Affiliations:** ^1^ Department of Biomedical Engineering Johns Hopkins University Baltimore Maryland USA; ^2^ Department of Radiation Oncology and Molecular Radiation Sciences Johns Hopkins University Baltimore Maryland USA

**Keywords:** clinical deployment, deep learning segmentation, pediatric brain cancer, radiotherapy

## Abstract

**Purpose:**

Radiation therapy (RT) of pediatric brain cancer is known to be associated with long‐term neurocognitive deficits. Although target and organs‐at‐risk (OARs) are contoured as part of treatment planning, other structures linked to cognitive functions are often not included. This paper introduces a novel automatic segmentation tool specifically designed for the unique challenges posed by pediatric patients undergoing brain RT, as well as its seamless integration into the existing clinical workflow.

**Methods and Materials:**

Images of 47 pediatric brain cancer patients aged 1 to 20 years old and 33 two‐year‐old healthy infants were used to train a vision transformer, UNesT, for the segmentation of five brain OARs. The trained model was then incorporated to clinical workflow via DICOM connections between a treatment planning system (TPS) and a server hosting the trained model such that scans are sent from TPS to the server, automatically segmented, and sent back to TPS for treatment planning.

**Results:**

The proposed automatic segmentation framework achieved a median dice similarity coefficient of 0.928 (frontal white matter), 0.908 (corpus callosum), 0.933 (hippocampi), 0.819 (temporal lobes), and 0.960 (brainstem) with a mean ± SD run time of 1.8 ± 0.67 s over 20 test cases.

**Conclusions:**

The pediatric brain segmentation tool showed promising performance on five OARs linked to neurocognitive functions and can easily be extended for additional structures. The proposed integration to the clinic enables easy access to the tool from clinical platforms and minimizes disruption to existing workflow while maximizing its benefits.

## 1 INTRODUCTION

Brain tumor is the leading cause of death among pediatric cancer patients with a prevalence of 5.14 cases per 100 000 children in the United States.^1^ While the use of radiation therapy (RT) is common for pediatric tumors, this treatment has been associated with neurocognitive impairment in intelligence quotient and academic performance.^2^ Studies have shown that the temporal lobe and hippocampus in particular, play a role in the formation and consolidation of new memories and that damage in these structures can lead to impairment in long‐term memory.^3^ The corpus callosum has also been linked to cognitive functions such as learning and memorization,^4^ and the frontal lobe to working memory, spatial processing, literacy, and numeracy.^5^, ^6^ Increasing radiation dose to these substructures is associated with worse performance in memory and processing speed.^7^ However, these substructures are not commonly contoured during RT planning which makes it difficult to minimize dose within these regions and mitigate the risk of memory and processing speed deficits. Manually segmenting these substructures would be time‐consuming and challenging to implement as part of the routine clinical workflow. This highlights the need to develop a user‐friendly tool that automatically segments brain substructures related to neurocognitive function and is integrated into the clinical workflow. This tool could also be used to perform a longitudinal analysis of anatomical changes over time and investigate how such changes would be associated with neurocognitive performance.

Convolutional neural networks (CNNs) have been widely used for brain segmentation and have shown high accuracy.^8^, ^9^, ^10^ More recently, vision transformers (ViT) have also been applied to the segmentation of brain organs at risk (OARs). These models were designed to handle sequences of data and can therefore capture long‐range dependencies between pixels or image regions.^11^ Methods combining ViT as encoder for feature extraction and CNN as decoder for segmentation have also been developed.^12^, ^13^, ^14^, ^15^ For example, Yu et al.^15^ proposed a nested model with three component hierarchical transformer as encoder paired with a convolution‐based decoder, the UNesT model. The method was applied to the segmentation of brain anatomy from T1‐weighted magnetic resonance imaging (MRI). An advantage of this method is that it preserves the inherent global self‐attention mechanism while establishing information exchange across patches through the hierarchical stacking of transformer encoders.

While such methods have shown state‐of‐the‐art segmentation performance, they were developed specifically for the adult brain and showed limitations when tested on pediatric data. Methods focusing specifically on pediatric brain segmentation have also been investigated but focused on specific applications targeting a single OAR.^16^, ^17^ Additionally, these methods do not segment structures proven to be associated with neurocognitive functions such as the frontal white matter, temporal lobe, and corpus callosum.

In this work, we propose an end‐to‐end framework integrated into the clinical workflow for the automatic segmentation of brain OARs related to neurocognitive functions in children undergoing RT for brain tumors. The autosegmentation of pediatric brains is a challenging task notably because they are still developing and only reach 90% of their adult volume by the age of 6^18^ which makes some key structures such as the white matter difficult to see and segment. Besides, the wider variability of brain volume and OARs shape within the pediatric population compared to the adult population makes the use of tools trained on adult data inadequate to accurately segment pediatric OARs. The proposed work aims at overcoming these challenges by developing a framework for pediatric brain OARs segmentation that seamlessly accepts MRI scans from diverse sources such as scanners, Picture Archiving and Communication Systems (PACS), or Treatment Planning Systems (TPS), automatically performs segmentation, and transmits the segmented output to any destination through a DICOM connection.

## 2 METHODS AND MATERIALS

### 2.1 Data

T1‐weighted brain MRI scans of 80 pediatric subjects from two different studies were used in the proposed work. Thirty‐three scans were obtained from a publicly available pediatric brain dataset^19^ and the other 47 from pediatric brain cancer patients treated with RT at the authors’ institution under the approval of the institutional review board. The scans of the public dataset were acquired from 2‐year‐old infants using a 1 T scanner (Philips Medical Systems, Best, Netherlands), and manual contours for 83 regions were made available, of which the corpus callosum, hippocampi, and frontal and temporal lobes were of interest to our study. The MRI scans from our institution were acquired with a 1.5 T scanner (Siemens Healthineers, Erlangen, Germany) from patients with ages ranging from 1 to 20 years old. Manual contours of the temporal lobes by radiation oncologists were available for all scans.

### 2.2 Data preprocessing

The publicly available SLANT framework^9^ was used to obtain contours for the cerebral white matter, hippocampi, and brainstem of all 47 cases acquired at our institution. This framework uses a 3D UNet architecture trained on 27 spatial locations separately, thus resulting in 27 3D UNet tiles each trained to segment a predefined brain region. The predictions of all network tiles are then fused into a consensus final segmentation using majority voting. The manual contours of the public dataset were leveraged and used as atlas to automatically label the corpus callosum and frontal lobe of the 47 cases from our institution using a multi‐atlas‐based segmentation (MABS) algorithm. The frontal white matter was then defined as the intersection of the frontal lobe and the white matter. All segmented labels were carefully reviewed and corrected as needed.

### 2.3 Vision transformer for brain OARs segmentation

**FIGURE 1:**
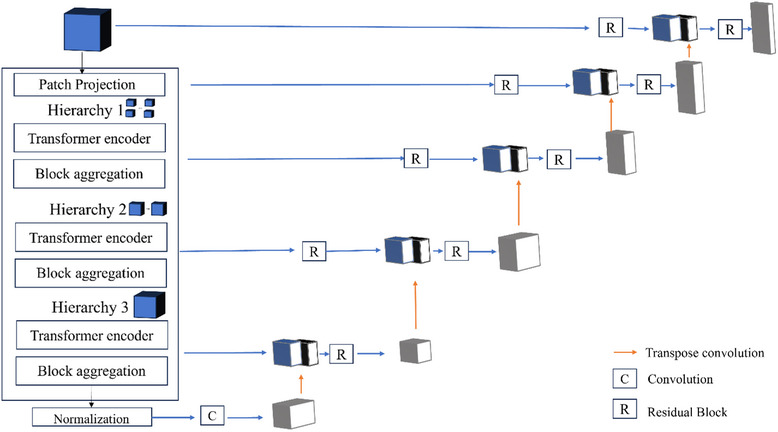
Illustration of the UNesT^15^ network architecture used in the proposed method.

The UNesT model^15^ was used in this work. As shown in Figure 1, the network architecture consists of a nested transformer‐based encoder and a convolution‐based decoder. The model was originally trained on a dataset of over 4000 adult brain MRI scans to segment 133 brain structures. The trained model and code were later released as part of the MONAI (Medical Open Network for Artificial Intelligence) Model Zoo.^20^ In the proposed work, we fine‐tuned this pre‐trained network on our smaller pediatric dataset to segment five OARs: the frontal white matter, corpus callosum, hippocampi, temporal lobes, and brainstem.

### 2.4 Model training

The UNesT model was implemented and trained using Python with Pytorch and MONAI. Among 80 MRI scans, 50 were used for training, 10 for validation, and 20 for testing. The network was trained for 300 epochs on NVIDIA RTX A5000 GPU on random patches of size 128 × 128 × 128 using the combined dice and cross‐entropy losses defined as follows:

$$\begin{array}{ccc} \;L^{Total} & = & L^{Dice}\;+\;L^{CE}=\;1-\frac{2\sum_{i}^{N}p_{i}g_{i}}{\sum_{i}^{N}p_{i}^{2}+\;\sum_{i}^{N}g_{i}^{2}}\\ & & -\frac{1}{N}\sum_{i}^{N}\sum_{c}^{C}g_{i,c}\;\log\left(p_{i,c}\right) \end{array}$$

where p is the predicted segmentation consisting of N voxels, g the ground truth, and C the total number of classes. Adam optimization was performed on batches with size of 1 and the learning rate of 10^−4^. The training data were augmented by random rotations along each axis with a probability of 0.1.

### 2.5 Integration to treatment planning system

The trained pediatric brain segmentation model was seamlessly integrated within clinical workflow. A framework to transfer MRI scans from any source (e.g., scanner, PACS, TPS) to the artificial intelligence (AI) server hosting the trained model, automatically segment the received scans, and send the output segmentation to any destination (most commonly TPS) via DICOM connection was developed. Communication between the source and the AI server was implemented using the Python package pydicom. Once the server receives the DICOM series, the segmentation pipeline is automatically triggered by a file monitoring system. The DICOM series is then converted to the Neuroimaging Informatics Technology Initiative (NIFTI) format for easier processing and used as input to the trained UNesT model to segment the OARs. The output network prediction is then converted from Python Numpy array to DICOM RT structure^21^ and automatically sent to a pre‐determined destination (RayStation in our setting) via DICOM connection.

### 2.6 Performance evaluation and statistical analysis

The performance of the segmentation method was evaluated in terms of dice similarity coefficient (DSC), 95th percentile of the Hausdorff distance (HD95), average symmetric surface distance (ASSD), and precision between the predicted and ground truth segmentations for twenty test cases. The median, min/max, and first (Q1) and third (Q3) quartiles were used to summarize the accuracy metrics. For performance comparison, we trained both the 3D UNet model and the 27 UNet network tiles as presented in the SLANT framework on our pediatric dataset. The latter was trained using the publicly available code from the authors and pre‐trained network. The Wilcoxon signed‐rank test was used to compare the performance of these three models against the ground truth of the test set.

## 3 RESULTS

**FIGURE 2:**
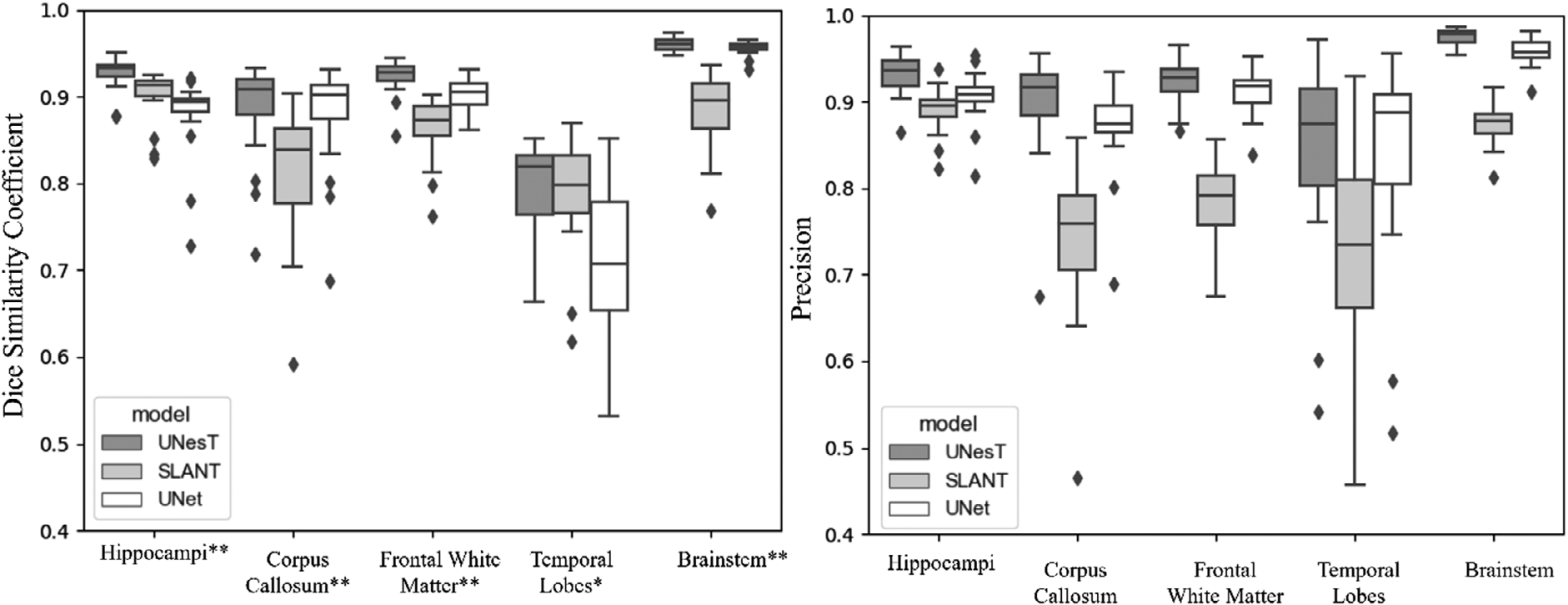
Dice similarity coefficient and precision distributions for the UNesT, SLANT, and 3D UNet models on the test dataset. * indicates structures showing significant difference in DSC (*p* < 0.05) between UNesT and UNet, and ** between UNesT and both SLANT and UNet based on Wilcoxon signed‐rank test.

The performance of the UNesT, SLANT, and UNet models on our test dataset is presented in Table 1, and Figure 2 illustrates the distribution of DSC values and precision for all three models on the test set. The Wilcoxon signed‐rank test on the DSC distributions revealed that the UNesT model performed significantly better (*p*‐value < 0.05) than the 3D UNet model for all structures. Significant differences were also found between UNesT and SLANT for all structures except the temporal lobes. A comparison with the UNesT model fine‐tuned on adult data is presented in Table S1.

**TABLE 1:** Segmentation performance of the UNesT, SLANT, and UNet models on the test dataset.

		DSC			HD95 (mm)			ASSD (mm)			Precision		
Model	OAR	Median	Min Max	Q1 Q3	Median	Min Max	Q1 Q3	Median	Min Max	Q1 Q3	Median	Min Max	Q1 Q3
UNesT	FWM	0.928	0.855 0.944	0.918 0.934	1.00	1.00 3.16	1.00 1.00	0.256	0.169 1.77	0.227 0.286	0.928	0.866 0.966	0.911 0.938
	Corpus callosum	0.908	0.719 0.933	0.879 0.919	1.00	1.00 4.24	1.00 1.00	0.241	0.209 1.00	0.230 0.345	0.916	0.675 0.956	0.885 0.931
	Hippocampi	0.933	0.877 0.951	0.922 0.936	1.00	1.00 1.41	1.00 1.00	0.174	0.107 0.422	0.155 0.188	0.936	0.865 0.963	0.919 0.947
	Temporal lobes	0.819	0.662 0.852	0.764 0.832	7.64	5.00 20.5	5.72 9.33	2.10	1.48 5.09	1.60 2.83	0.875	0.542 0.971	0.803 0.914
	Brainstem	0.960	0.947 0.974	0.954 0.966	1.00	1.00 1.00	1.00 1.00	0.196	0.123 0.265	0.151 0.222	0.978	0.953 0.987	0.969 0.981
SLANT	FWM	0.873	0.762 0.902	0.855 0.890	2.45	1.41 19.4	2.24 5.39	0.826	0.419 3.11	0.690 1.18	0.791	0.675 0.857	0.757 0.815
	Corpus callosum	0.839	0.592 0.904	0.777 0.864	2.64	1.41 33.6	2.00 4.60	0.996	0.445 5.98	0.916 1.62	0.759	0.456 0.858	0.706 0.791
	Hippocampi	0.914	0.829 0.925	0.901 0.918	1.00	1.00 1.73	1.00 1.10	0.398	0.203 1.47	0.305 0.562	0.895	0.822 0.938	0.883 0.903
	Temporal lobes	0.798	0.618 0.870	0.766 0.833	8.88	5.00 27.4	6.16 12.1	2.39	1.56 7.58	2.17 3.13	0.735	0.457 0.930	0.662 0.809
	Brainstem	0.896	0.768 0.937	0.862 0.914	2.81	1.00 14.6	1.93 5.94	0.949	0.409 2.75	0.659 1.08	0.878	0.812 0.916	0.864 0.886
UNet	FWM	0.905	0.861 0.931	0.891 0.915	1.41	1.00 3.00	1.00 2.00	0.593	0.284 1.67	0.388 0.739	0.918	0.839 0.952	0.899 0.924
	Corpus callosum	0.901	0.687 0.931	0.874 0.913	1.00	1.00 5.20	1.00 1.41	0.293	0.209 1.03	0.256 0.382	0.875	0.689 0.934	0.865 0.895
	Hippocampi	0.893	0.728 0.921	0.883 0.897	1.00	1.00 3.74	1.00 1.00	0.275	0.211 0.855	0.255 0.294	0.908	0.815 0.954	0.901 0.917
	Temporal lobes	0.707	0.531 0.852	0.654 0.778	8.74	5.39 22.11	7.23 10.2	2.62	1.64 5.63	2.02 3.17	0.888	0.517 0.955	0.804 0.909
	Brainstem	0.957	0.931 0.966	0.954 0.960	1.00	1.00 1.00	1.00 1.00	0.207	0.151 0.289	0.191 0.230	0.957	0.912 0.981	0.951 0.969

Abbreviation: FWM, frontal white matter.

The mean ± SD segmentation run time of the proposed method evaluated on the test dataset was 1.8 ± 0.67 s on a NVIDIA RTX A5000 GPU. This included the time required to load the trained model, preprocess the input volume, and perform the patch‐based inference using a sliding window of size 128 × 128 × 128 with 50% overlap between patches.

**FIGURE 3:**
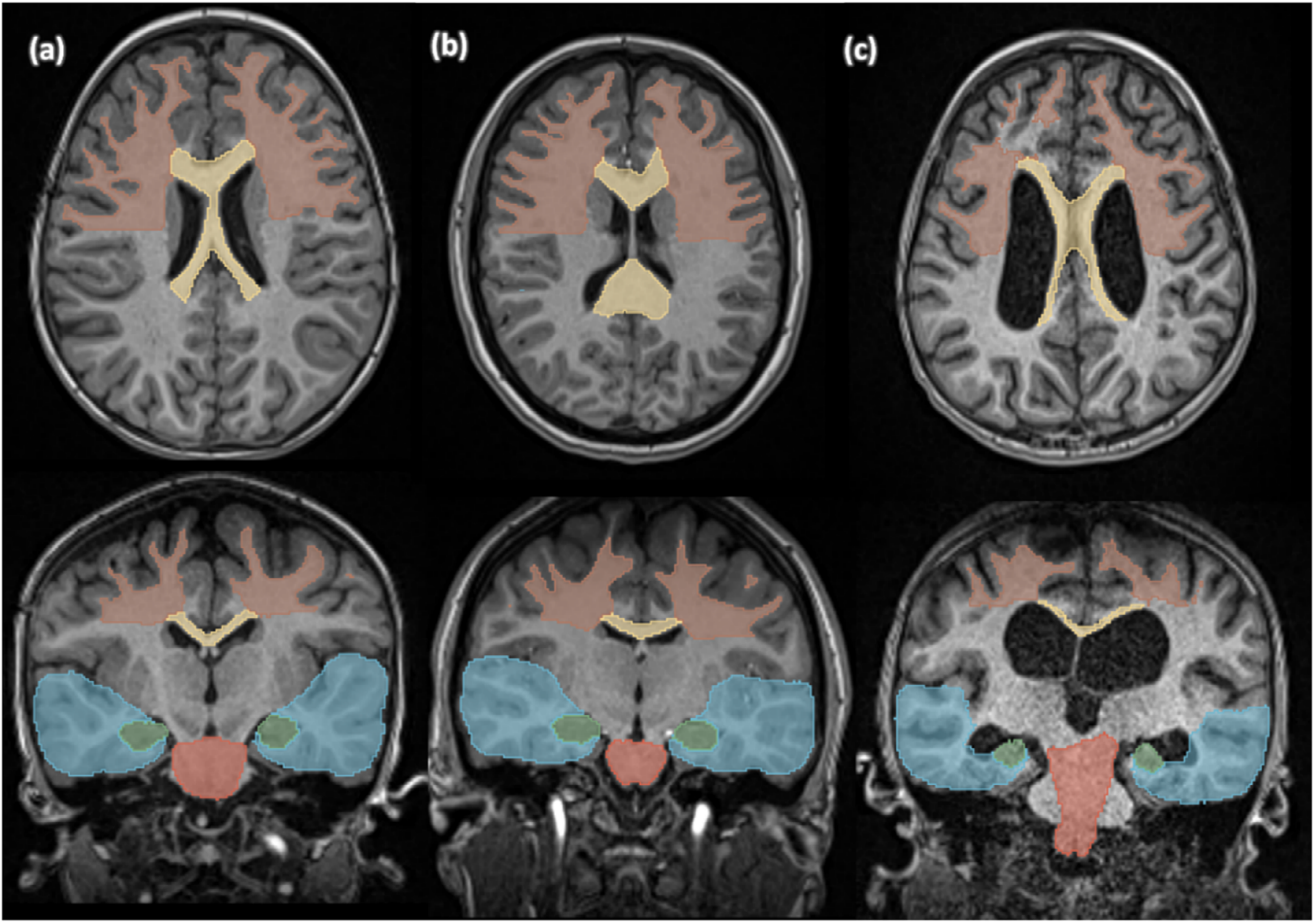
Example segmentations showing the (a) highest, (b) median, and (c) lowest performance of the UNesT model among test cases. The frontal white matter is shown in brown, corpus callosum in yellow, hippocampus in green, temporal lobe in blue, and brainstem in red.

Figure 3 illustrates examples of predicted segmentations from our test set. Figure 3a represents an axial and a coronal slice of the scan on which the UNesT achieved the highest average DSC of 0.934, Figure 3b represents the median performance with 0.912, and Figure 3c the lowest with 0.858. This figure illustrates the variability in the size and shape of the ventricles included in our test set.

## 4 DISCUSSION

Evaluating the performance of UNesT, SLANT, and 3D UNet revealed that the UNesT model consistently outperformed the other two models across four of the five brain regions and for all metrics evaluated. The Wilcoxon signed‐rank test was used to compare these methods. In comparing UNesT to the original 3D UNet, UNesT resulted in a significantly higher DSC for all five OARs. The comparison of UNesT and SLANT showed that UNesT achieved significantly higher DSC for four of the five OARs: the frontal white matter, corpus callosum, hippocampi, and brainstem. No significant difference was observed for the temporal lobes. Overall, these results highlight the effectiveness of UNesT in achieving more accurate and robust segmentation across a range of brain regions, emphasizing its potential for additional planning structures. The superiority of UNesT compared to SLANT and UNet can be explained by their difference in architectures. Indeed, while SLANT and UNet are CNNs, UNesT combines a transformer as an encoder, able to handle sequences of data, with a convolution‐based decoder, thus capturing not only local image features but also long‐range dependencies between voxels and leading to improved segmentation performance over CNNs.

The ground truth labels for the training, validation, and testing datasets used in the proposed work were obtained using a semi‐automatic approach by manually correcting labels obtained from a MABS algorithm. This choice was made due to the difficulty of manually contouring our target structures, in particular, the frontal white matter. Indeed, the frontal white matter is challenging to manually delineate due to its complex shape. Consequently, we instead chose to adopt a semi‐automatic approach for four of the five structures. The remaining structure, the temporal lobes, was readily accessible for all test cases since it is commonly contoured as part of treatment planning. We therefore employed manual contours for this region. Compared to the other structures studied in this work, the temporal lobe is arguably one of the most challenging to segment due to the lack of clear anatomical features marking its boundaries. Neural networks are very efficient at detecting edges, which is crucial for segmentation tasks. The absence of distinct edges for the temporal lobe could explain why the model achieved the lowest DSC on this particular structure.

The contributions of this work are threefold. First, we proposed a pediatric brain segmentation neural network achieving state‐of‐the‐art performance for five OARs and release the code and trained model publicly.^a^ We utilized an existing neural network model^15^ which was originally trained for the segmentation of 133 brain structures, primarily consisting of gyri and sulci, based on adult T1‐weighted MRI datasets. Our approach involved fine‐tuning the released pre‐trained model on our smaller pediatric dataset, focusing specifically on structures relevant to tissue sparing for neurocognitive functions. While we only trained it to segment five structures, the model can easily be configured to handle any number of structures. We employed this model as a proof of concept within our broader automatic segmentation framework, although any suitable model could be used in its place. Although tools for the segmentation of adult brain have been released, to the best of our knowledge there exists no publicly available tool specifically developed for the simultaneous segmentation of pediatric frontal white matter, corpus callosum, hippocampi, temporal lobes, and brainstem. These structures were chosen due to their importance in neurocognitive assessment. Our goal was to develop an automatic segmentation tool that can be used in the RT planning process to reduce dose to substructures associated with neurocognitive significance and used for a longitudinal study aiming at analyzing anatomical changes in brain structures related to cognitive functions in children undergoing brain radiotherapy. Direct comparison of the proposed segmentation method with other publicly available brain segmentation tools is therefore challenging. Out of the five structures studied, only the hippocampi and brainstem are commonly supported by other tools and can be compared. For example, a public adult brain segmentation tool, MALPEM,^22^ reported a DSC of 0.869 for the hippocampi and 0.940 for the brainstem with a run time of 1–2 h on eight cores CPU and 10 h on single‐core CPU. Our fine‐tuned pediatric UNesT, although trained and tested on fewer subjects, achieved comparable accuracy of 0.933 and 0.960 for these two structures respectively, and a mean ± SD run time of 1.8 ± 0.67 s. Integrating this tool into clinical workflow will allow fast and streamlined processing of patient scans at each time point.

The second contribution is the development of a simple yet efficient framework to integrate any deep learning segmentation model to clinical workflow. Publicly available tools often require a specific environment which makes their integration to clinic challenging and require user interaction via the command line. Our method has the benefit of requiring very little to no user interaction, that is, a scan is sent from any source, processed, and sent to any destination. The feasibility of the approach was demonstrated using the UNesT model due to its status as one of the most recent best‐performing networks as shown in the original paper^15^ where rigorous comparisons to state‐of‐the‐art methods such as nnUNet, nnFormer, and SwinUNETR across various public datasets were performed.

Finally, the segmentation framework consists of a deep learning network that operates without the need for extensive preprocessing, such as N4 correction^23^ or registration to the MNI space,^24^ resulting in significantly enhanced processing speed. The speed of the proposed segmentation module significantly outpaces other publicly available tools with a mean ± SD of 1.8 ± 0.67 s over the 20 test cases, compared to FreeSurfer,^25^ which takes approximately 10 h, or SLANT, which requires around 15 min for scan processing. However, we acknowledge that this comparison may not be entirely equitable, as SLANT and FreeSurfer handle a considerably larger number of structures compared to our proposed approach. Nevertheless, the advantage of our method lies in the utilization of a deep learning model with GPU acceleration, which suggests that it would likely maintain its computational efficiency even when tasked with segmenting a greater number of structures. Future work will include the collection of additional training and testing data with ground truth segmentations for additional brain structures.

## 5 CONCLUSION

In this work, a framework for the automatic segmentation of pediatric brain OARs related to neurocognitive functions was proposed. The framework leverages DICOM connections to transfer scans from any source (e.g., scanner, PACS, TPS) to an AI server hosting the trained segmentation model, segment the received scans, convert the predicted segmentation to DICOM RT structure, and send it to a pre‐determined location. The feasibility of the method was demonstrated using RayStation as a source and the UNesT network architecture trained on five brain structures. The speed and accuracy of the proposed method make it a valuable tool for normal tissue sparing RT planning and the longitudinal analysis of anatomical variations in the segmented structures during and after RT of pediatric brain cancer patients.

## AUTHOR CONTRIBUTIONS

All the authors significantly contributed to the design of the work, image data acquisition and interpretation, deep learning model development and evaluation as well as the manuscript writing and final approval of the version to be published. Lina Mekki and Junghoon Lee curated image data, developed and trained the proposed deep learning model, incorporated the tained model into clinical setting, and performed evaluation. Sahaja Acharya and Matthew Ladra provided clinical feedback for data collection, ground truth manual segmentations, and assessed the segmentation quality and clinical workflow.

## CONFLICT OF INTEREST STATEMENT

The authors declare no conflict of interest.

## Supporting information

Supporting Information

## Data Availability

A sample image data is provided in our code repository (https://github.com/JHU‐MICA/JACMP2024_PedsBrainSeg). Institutional image data are not available for sharing, but public pediatric brain image data^19^ used in this study are available for research from http://www.brain‐development.org.

## Footnotes

a
https://github.com/JHU‐MICA/JACMP2024_PedsBrainSeg
