# Supplementary material for: Deep learning segmentation of organs‐at‐risk with integration into clinical workflow for pediatric brain radiotherapy
Source: J Appl Clin Med Phys. 2024 Feb 19;25(3):e14310. doi: 10.1002/acm2.14310 (PMC10930010; doi:10.1002/acm2.14310)
Supplement: Supplementary file 1 — Supporting Information [file ACM2-25-e14310-s001.pdf]

Supplemental materials

S.1. Comparison of adult-trained and pediatric-trained UNesT.

To highlight the need of a tool specifically trained on pediatric data for pediatric brain OAR segmentation, the original UNesT model was trained on an adult dataset for the segmentation of the frontal white matter, corpus callosum, hippocampi, temporal lobes, and brainstem.

Data

Fifty adult MRI scans were collected from 50 brain radiotherapy patients. These adult cases originally did not include the structures studied in this paper. To obtain the ground truth segmentations, the fine-tuned pediatric UNesT was used to create initial segmentations followed by manual corrections. Since the pediatric model was trained on patients aged 1 to 21 years old, and that pediatric brains reach 90% of their adult volume by the age of 6 [19], this was a reasonable initialization for the ground truth generation. A test subset consisting of 9 infants with ages ranging from 1 to 6 years old drawn from the test set was used for DSC comparison between the adult and pediatric UNesT models.

Model Training

The adult UNesT was trained using the same parameters as the pediatric model. The network was trained for 300 epochs on NVIDIA RTX A5000 GPU on random patches of size 128×128×128 using the combined dice and cross entropy losses defined in Methods and Materials – Model Training; Adam optimization was performed on batches with size of 1 and the learning rate of 10^{-4}; the training data were augmented by random rotations along each axis with a probability of 0.1.

Results

The median DSC, minimum and maximum, and first and third quartiles for the pediatric and adult models are displayed in Table S.1. These results show that the pediatric UNesT outperformed the adult UNesT on four of the five structures studied especially when the brain is smaller than normal adult brain (i.e. for young children).

Table S.1: Segmentation performance of the pediatric and adult UNesT models on a test subset MRIs of nine infants aged 1 to 6 years old. Bold highlights the best performance for each OAR. FWM: frontal white matter.

Model	DSC	OARs				
		FWM	Corpus Callosum	Hippocampi	Temporal Lobes	Brainstem
Peds UNesT	Median	**0.928**	**0.906**	**0.935**	0.829	**0.957**
	Min	0.894	0.843	0.877	0.696	0.951
	Max	0.943	0.922	0.951	0.850	0.972
	Q1	0.923	0.883	0.928	0.770	0.955
	Q3	0.934	0.916	0.938	0.833	0.966
Adult UNesT	Median	0.900	0.897	0.906	**0.841**	0.940
	Min	0.866	0.798	0.848	0.711	0.925
	Max	0.918	0.912	0.914	0.870	0.954
	Q1	0.886	0.863	0.901	0.831	0.932
	Q3	0.909	0.903	0.912	0.847	0.946
